# An Industrial and Sustainable Platform for the Production of Bioactive Micronized Powders and Extracts Enriched in Polyphenols From *Olea europaea* L. and *Vitis vinifera* L. Wastes

**DOI:** 10.3389/fnut.2020.00120

**Published:** 2020-08-21

**Authors:** Annalisa Romani, Margherita Campo, Silvia Urciuoli, Giulia Marrone, Annalisa Noce, Roberta Bernini

**Affiliations:** ^1^PHYTOLAB (Pharmaceutical, Cosmetic, Food Supplement, Technology and Analysis), DiSIA, University of Florence, Florence, Italy; ^2^PhD School of Applied Medical, Surgical Sciences, University of Rome Tor Vergata, Rome, Italy; ^3^UOC of Internal Medicine-Center of Hypertension and Nephrology Unit, Department of Systems Medicine, University of Rome Tor Vergata, Rome, Italy; ^4^Department of Agriculture and Forest Sciences (DAFNE), University of Tuscia, Viterbo, Italy

**Keywords:** *Olea europaea* L., *Vitis vinifera* L., agro-industrial wastes, circular economy, sustainable extraction, polyphenol-enriched extracts, micronized powders, biological activities

## Abstract

In the last few years, literature data have reported that health status is related to the consumption of foods rich in polyphenols, bioactive compounds found in the plant world, in particular in vegetables and fruit. These pieces of scientific evidence have led to an increase in the demand for functional foods and drinks enriched in polyphenols, so that plant materials are more and more requested. The availability of food and agricultural wastes has adverse effects on the economy, environment, and human health. On the other hand, these materials are a precious source of bioactive compounds as polyphenols. Their recovery and reuse from wastes are according to the circular economy strategy, which has introduced the “zero waste concept.” However, the process is convenient from an economic and environmental point of view only if the final products are standardized and obtained using sustainable and industrial technologies. In this panorama, this paper describes an industrial and sustainable platform for the production of micronized powders and extracts enriched in polyphenols from *Olea europaea* L. and *Vitis vinifera* L. wastes that are useful for food, cosmetics, and pharmaceuticals sectors. The platform is based on drying plant materials, extraction of polyphenols through membrane technologies with water, and, when necessary, the concentration of the final fractions under vacuum evaporation. All powders and extracts were characterized by high-performance liquid chromatography–diode array detector–mass spectrometry analysis to define the qualitative and quantitative content of bioactive compounds and insure their standardization and reproducibility. The chromatographic profiles evidenced the presence of secoiridoids, flavones, flavonols, anthocyanins, hydroxycinnamic acids, catechins, and condensed tannins. An overview of the biological activities of the main polyphenols present in *Olea europaea* L. and *Vitis vinifera* L. powders and extracts is reported because of biomedical applications.

## Introduction

For many years, scientific research has shown the existence of the close link between the consumption of certain foods and health status. This induces consumers to believe that their health can be controlled, at least to a certain extent, through a careful selection of foodstuffs and their components. In this context, particular attention is paid both to food quality, functional properties, and secondary metabolites of plant foods. The volatile molecules on which depends the aroma of food and especially to the water-soluble secondary active metabolites are often responsible for food taste, stability, and its healthy properties. As far as the prevention of certain pathologies is concerned, research has demonstrated how some specific components can contribute to an improvement in overall health. In other words, it has been observed a growing interest from consumers toward functional ingredients that, in varying ways, can counteract the symptoms of some illnesses or contribute to their prevention (1). In fact, one of the main reasons for the enhanced consumption of functional food on a global level is the increased awareness of the role of a balanced diet in maintaining a healthy status and in preventing chronic degenerative non-communicable diseases (CDNCDs) caused by various factors, among these an unbalanced diet (2, 3). CDNCDs include cardiovascular (CV) disease, arterial hypertension, cancer, chronic kidney disease (4, 5), metabolic syndrome, obesity (6), and diabetes mellitus (7–10). Moreover, on an institutional level, the ever-increasing public health expenditure for the treatment of CDNCDs calls for a preventive intervention that can be implemented thanks also to the consumption of functional food. In fact, these foods have demonstrated their efficacy in the prevention of CV diseases, the main cause of death in developed countries (11–14). The demand for functional food is positively influenced by the fact that consumers consider these products as a more practical and natural therapeutic alternative, with fewer adverse effects compared with pharmacological treatment (15). Furthermore, the increase of lifespan associated with a greater probability of CDNCDs development represents a further element in the spread of functional food markets, especially in Europe and North America (16, 17).

In this regard, a relevant role is played by polyphenols, natural compounds found in vegetable foodstuffs showing a variety of beneficial effects for human health. Since the 1990s, the interest for these compounds has been increasing thanks to a large number of scientific studies demonstrating their effectiveness in the correct maintenance of health status and in particular in treating diabetes mellitus, CV diseases, dyslipidemia, and cancer, along with a rising number of applications in the food, beverage, and pharmaceutical sectors. In a recent report (18), the size of the global polyphenols market is expected to reach USD 2.08 billion by 2025, expanding at a compound annual growth rate of 7.2% during this period.

In consideration of these data, it is very important to have natural sources to obtain polyphenols. Wastes and by-products deriving from many agricultural and food sectors offer a solution to satisfy the polyphenol market and present a good opportunity to reduce their negative impacts on both environment and economy related to their disposal (19, 20). The reuse and then the valorization of the agro-industrial wastes represent an important goal for the preservation and support of a sustainable ecosystem and effective production (21). The agricultural waste recycling perfectly fits with the “zero waste concept” introduced by the circular economy strategy (22, 23), and it is based on environmentally friendly processes for recovering polyphenols on both laboratory and industrial scale showing biological activities (24–27).

A remarkable innovation of process consists of obtaining extracts naturally enriched in bioactive compounds (28–31). Advanced plant material drying techniques, micronization, extraction, and membrane purification technologies go in this direction, contributing to increasing the added value of the final products and their industrial potential in agronomic, food, cosmetic, and pharmaceutical sectors.

Two typical agricultural productions of the Mediterranean area are extra virgin olive oil (EVOO) and wine. The daily consumption of these foods is responsible for a variety of beneficial health effects (31–35). The main product of olive tree cultivation (*Olea europaea* L.) is EVOO; however, this production is associated with a large number of wastes and by-products, mainly olive pulp, and olive leaves. These wastes represent an important source of active phenolic compounds; in fact, only a small part of these ends up in oil (<0.5%); the remaining part is present in the coproducts (36).

Similarly, winemaking of *Vitis vinifera* L. produces wastes consisting of solid residues (pomace), lees, and wastewater. White wine vinification, carried out without maceration of skins in the must, produces stalks and pomace; red wine vinification, performed with skin maceration, leads immediately to the formation of steams and after the period of pomace maceration. A considerable amount of organic solid waste is produced during the crushing and pressing processes and wine clarification. After the pressing to obtain juice/must, pomace contains mainly grape skins, seeds, and some pulp residue. It is estimated that only in Italy, 0.1–0.3 million tons/year of seeds are produced from the winemaking process (37).

In this panorama, this manuscript describes an industrial and sustainable platform for the production of micronized powders and extracts enriched of polyphenols from *Olea europaea* L. and *Vitis vinifera* L., dried vegetal tissues, and liquid and solid wastes. All powders and extracts were characterized by HPLC-DAD-MS analysis to define the qualitative and quantitative content of polyphenols and insure their standardization and reproducibility. In particular, this work describes both innovative processes and products from Olea and Vitis obtained as micronized powders and extracts, not yet reported in scientific works for characterization and promising biological activities.

To provide an overview that analyzes both a sustainable platform for extraction of powders and its potential effects on human health, in this paper, we explored literature data that support beneficial effects derived from *Olea europaea* L. and *Vitis vinifera* L. (38). For example, the effect of one of the principal phenolic compounds of EVOO, oleocanthal (OLC), was described for the first time in 2005 by Beauchamp et al. (39). This component gives to the EVOO its characteristic pungent taste, and this flavor is like that induced by the assumption of ibuprofen, a nonsteroidal anti-inflammatory drug. Therefore, these authors investigated the anti-inflammatory action exerted by OLC in humans, demonstrating that the enzymatic inhibition of OLC is dose-dependent and it would be more effective than that induced by ibuprofen drug. In fact, an OLC concentration of 25 μM can inhibit the COX activity from 41 to 57% vs. 25 μM of ibuprofen that inhibits COX from 13 to 18%, respectively (39). Subsequently, other clinical trials showed that chronic low doses of ibuprofen could also exert anti-cancerogenic and anti-thrombotic effects (40, 41) by reducing the chronic low-grade inflammatory state, a condition commonly present in the CDNCDs.

Concerning health effects of *Vitis vinifera* L., the first observation that highlighted its health potential is the “French paradox” (42), in which the authors concluded that daily alcohol consumption, ranging from 20 to 30 g, was able to reduce CV mortality up to 40%. The authors ascribed this revolutionary founding to the hemostatic activity carried out by wine. Subsequently, over the years, further studies have been carried out to confirm the beneficial effects induced by the consumption of wine and by wine sector derivatives (43–45).

## Materials and Methods

### Chemicals and Plant Materials

Reagents, solvents, and chemicals were supplied by Sigma-Aldrich (Milan, Italy). Tyrosol, oleuropein aglycone, OLC, caffeic acid, gallic acid, verbascoside, luteolin 7-O-glucoside, quercetin 3-O-glucoside, quercetin 3-O-glucuronide, kaempferol 3-O-glucoside, *cis*-resveratrol, *trans*-resveratrol glucoside, catechin, epicatechin, catechin dimer B3, catechin dimer B6, malvidin 3-O-glucoside, cyanidin 3-O-glucoside, petunidin 3-O-glucoside, peonidin 3-O-glucoside, and delphinidin 3-O-glucoside used as standards were supplied by Extrasynthèse (Genay, France). Pure hydroxytyrosol (HT) was synthesized in the laboratory, as reported in the literature (46).

*Olea europaea* L. samples and relative liquid and solid wastes were obtained from Frantoio, Leccino (Florence, Italy), and Seggianese (Grosseto, Italy) cultivars. *Vitis vinifera* L. samples, red wine solid residue production, grapes marcs, grapes peels, and seeds were obtained from Sangiovese (Siena, Italy), Sagrantino (Perugia, Italy), and Merlot (Latina, Italy) cultivars. Commercial extracts of grape seeds and leaves were purchased from Sochim (Milan, Italy) and Indena (Milan, Italy), respectively.

### Production of Micronized Powders and Extracts From *Olea europaea* L.

The industrial platform is based on drying of plant materials, water extraction of polyphenols and secoiridoids, concentration through membrane technologies with water, and, when necessary, a final concentration of the single fractions under vacuum evaporation.

The drying of plant materials was performed with dryer cell–hot air input from bottom–up (Dermasole Srl, Italy) under humidity and temperature control. Finally, a sanitization with ozone and UV soft radiations was carried out. Olea leaves and oil-free olive destoned pulps, after EVOO plant production, were dried using dryer cell–hot air input from bottom–up technologies, as reported in Table 1. The process for the leaves provided the temperature of 35°C for 12 h; the oil-free olive destoned pulps were dried for 12 h at the temperature of 40°C and 24 h at 35°C until complete drying (max 5% activity water). From 100 kg of product, the dry product was obtained in yields 45–72%.

**Table 1:** Technologies used for drying Olea leaves and oil-free olive destoned pulps of the dried product.

**Technology**	**Plant material**	**Yield (%)**
Dryer cell–Hot air input from bottom up	Olea leaves	72
Dryer cell–Hot air input from bottom up	Oil-free olive destoned pulps	45

Membrane technologies obtained industrial purification and bioactive extract concentrations. The process consists of the following steps carried out in sequence: (a) provision of the plant materials; (b) obtaining of Olea leaves or oil-free olive destoned pulps after EVOO plant productions; (c) acidification of the vegetal material to a pH = 2.5–4.0; (d) extraction at room temperature with an aqueous solvent using an electrical pneumatic extractor; (e) microfiltration, ultrafiltration, nanofiltration, and reverse osmosis of the resulting solution; (f) concentration of the fractions under vacuum by using a scraper evaporator series (C&G Depurazione Industriale Company) combined with a heat pump (25, 28, 47). The concentrate fraction samples rich in HT were obtained after preparative liquid chromatography (LC). The EVOO minor polar compounds fraction enriched in secoiridoids and polyphenols was obtained from (a) EVOO hydroalcoholic (EtOH/H_2_O = 70:30) ultrasound extraction and treatment; (b) microfiltration, ultrafiltration, and nanofiltration; (c) acidification to a pH = 2.5–4.0; and (d) LC purification. Using these technologies and purification systems, the yield of this process does not exceed 0.02%. All processes are registered in the EU or International Patent Cooperation Treaty (47, 48).

### Production of Micronized Powders and Extracts From *Vitis vinifera* L.

The industrial platform is based on drying of plant materials, hydroalcoholic extraction of polyphenols, concentration through membrane technologies, and, when necessary, concentration of the fractions from under vacuum evaporation.

Vitis leaves, grape marc, peel, and seeds were dried using different technologies as reported in Table 2. From 100 kg of fresh material, the dried product was obtained in yields 33–98%.

**Table 2:** Technologies for drying Vitis leaves, grape marc, peel, seeds, and yields of the dried product.

**Technology**	**Starting material**	**Yield (%)**
Heat carrier oven from cogenerator	Vitis leaves	66
Heat carrier oven from cogenerator	Vitis leaves	66
Drying cabinet for medicinal species	Vitis leaves	33
Heat carrier oven from cogenerator	Grape marc (70% peel; 30% seeds)	45
Dryer cell–Hot air input from bottom up	Grape marc (70% peel; 30% seeds)	80
Dryer cell–Hot air input from bottom up	Grape peel	72
Dryer cell–Hot air input from bottom up	Grape seeds	98

The drying of plant materials was performed with dryer cell–hot air input from bottom–up (Dermasole Srl, Italy) under humidity and temperature control; a sanitization with ozone and UV soft radiation was the last step.

The industrial plant for aqueous and hydroalcoholic extraction and tannin-enriched fraction productions of grape seeds by membrane technologies was previously described (24, 37).

### Chemical Characterization of Micronized Powders and Extracts

The qualitative and quantitative content of powders and extracts were determined by HPLC-DAD-MS analysis. An HP 1200 LC (Agilent Technologies, Palo Alto, CA, USA) equipped with an analytical column Lichrosorb RP18 250 × 4.60 mm i.d, 5 μm (Merck Darmstadt, Germany) was used. The eluents were H_2_O adjusted to pH = 3.2 with HCOOH (solvent A) and CH_3_CN (solvent B). A four-step linear solvent gradient was used, starting from 100% of solvent A up to 100% of solvent B, for 88 min at a flow rate of 0.8 ml min^−1^. Polyphenols found in the extracts were identified by comparing retention times and UV/Vis spectra with those of the authentic standards (see *Chemicals and Plant Materials*). Each compound was quantified at the selected wavelength (240, 280, 330, and 350 nm) using a five-point regression curve (49). For the analyses of anthocyanins, a Luna column C18 250 × 4.60 mm, 5 μm (Phenomenex) was used. The eluents were H_2_O adjusted to pH = 1.8 with HCOOH (solvent A) and CH_3_CN (solvent B). A multistep linear solvent gradient was used, starting from 95% A up to 100% B for 24 min at a flow rate of 0.8 ml min^−1^. Anthocyanins were identified by comparing retention times and UV/Vis spectra with those of the authentic standards (see *Chemicals and Plant Materials*). The quantification of the individual compounds was performed at 520 nm using a five-point regression curve built with standard solutions of malvidin 3-O-glucoside and applying the correction of the molecular weights.

## Results and Discussion

### Micronized Powders and Extracts Enriched in Polyphenols and Secoiridoids From *Olea europaea* L. Wastes

#### Production and Chemical Characterization

**Figure 1:**
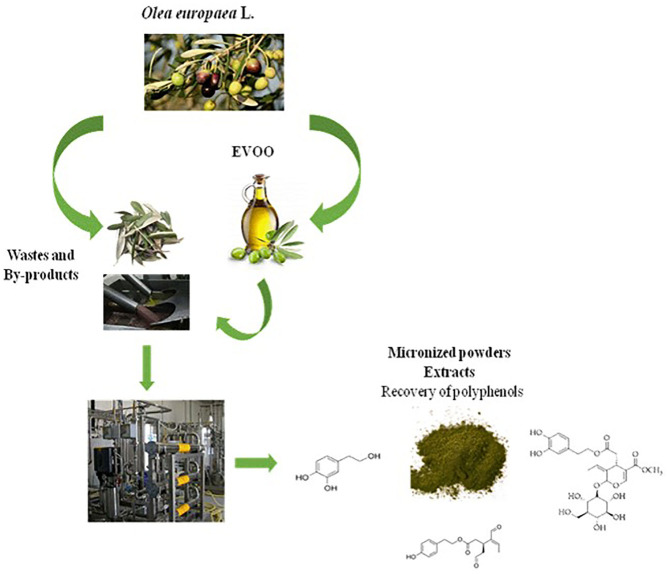
Production of EVOO and valorization of Olea wastes.

Olive oil wastes are profitable resources; their reuse and valorization open up interesting agronomic, environmental, and economic opportunities by a parallel market to that of EVOO production, as depicted in Figure 1.

In this paper, a platform based on new sustainable technologies to produce polyphenol-enriched powders and extracts from *Olea europaea* L. wastes was described, in particular from Olea leaves and oil-free olive destoned pulps, after EVOO plant production.

Olea leaves and oil-free olive destoned pulps, after EVOO plant production, were dried using dryer cell–hot air input from bottom–up technologies, as reported in Table 1 (see *Materials and Methods*). The HPLC-DAD-MS analysis of the powders obtained after oil pressing (AP) and AP drying (AD) was reported in Table 3. Recent studies have demonstrated that the phenolic content of samples deriving from Olea wastes is stable over time after drying (50).

**Table 3:** HPLC-DAD-MS analysis of oil-free olive pulp powder after oil pressing (AP) and AP drying (AD).

**Compound**	**AP**	**AD**
	**mg/Kg**	**mg/Kg**
Hydroxytyrosol	(6.9 ± 0.3) × 10^3^	(1.27 ± 0.05) × 10^3^
Tyrosol	(3.7 ± 0.1) × 10^3^	(4.2 ± 0.2) × 10^2^
*Total polyphenols*	(1.06 ± 0.04) × 10^4^	(1.69 ± 0.07) × 10^3^

As shown in Table 3, the HT content is higher in AP than that in AD; for this characteristic, AP is used for nutraceutical purposes even if their production requires a lot of energy and high drying times. The drying of the samples after the EVOO production and the elimination of water (AD) allows the enhancement and use of vegetable water rich in active compounds directly in the extraction phase; moreover, these can be used for food, feed, and cosmetic production.

The dried oil-free olive pulp powder is an innovative product, enriched in fibers and HT with antioxidant properties, useful for feed and food formulations, and for bakery production.

As an implementation of a methodology described at a laboratory scale (51, 52), an innovative process has been developed at an industrial level by using physical technologies defined as best available technology and recognized by the Environmental Protection Agency (28, 47). The process is based on membrane technologies applied to aqueous extracts obtained in a pneumatic extractor and then purified by filtration. It consists of the steps described in the materials and methods section. The integrated systems of the membrane, characterized by different molecular weights with cut-off and filtration degrees, are strategic because they allow us to obtain fractions containing precious polyphenols as HT and oleuropein (OLE). Finally, the concentration under vacuum by using a scraper evaporator series combined with a heat pump increases the concentration of polyphenols and allows us to obtain purified active compounds belonging to different subclasses.

For each extract obtained from the industrial plant, HPLC-DAD-ESI/MS analysis is performed to identify and quantify polyphenolic classes, highlighting the content of over 90% of HT and derivatives among them (28). As a result, three commercial products were obtained through this process: a liquid extract, a solid extract after membrane purification and vacuum concentration, and an extract after LC. As reported in Table 4, all extracts show a standardized content of polyphenols and a high concentration of HT. The liquid extracts can be marketed as either concentrated solutions or powder after drying.

**Table 4:** Polyphenolic composition of different commercial extracts obtained from olive solid wastes.

**Compound(s)**	**Liquid extract**	**Solid extract**	**Extract after LC**
	**mg/ml**	**mg/g**	**mg/g**
Hydroxytyrosol glycol	0.0032 ± 0.0001		
Hydroxytyrosol	0.026 ± 0.001	(1.15 ± 0.04) × 10^2^	(9.9 ± 0.4) × 10^2^
Tyrosol and derivatives	0.0153 ± 0.0006	(3.45 ± 0.13) × 10	2.23 ± 0.09
*Total polyphenols*	0.044 ± 0.002	(1.50 ± 0.05) × 10^2^	(9.9 ± 0.5) × 10^2^

**Figure 2:**
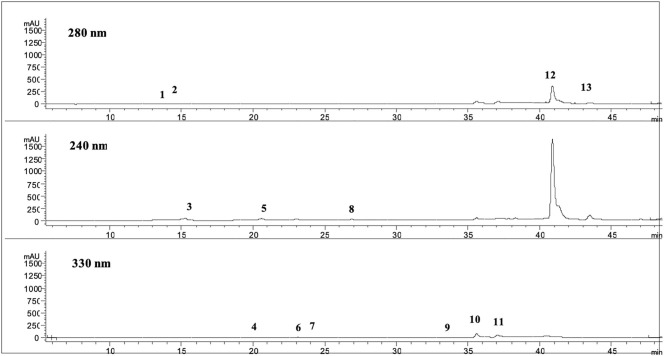
Chromatograms at 280, 240, and 330 nm of *OLP1*. 1. Hydroxytyrosol glucoside; 2. Hydroxytyrosol (HT); 3. Demethyl elenolic acid diglucoside; 4. Caffeic acid derivative; 5. Oleoside; 6. *p*-Coumaric acid; 7; Chlorogenic acid; 8. Derivative list oil; 9. β-OH-verbascoside; 10. Verbascoside; 11. Luteolin 7-O-glucoside, 12. Oleuropein (OLE); 13. Oleuropein aglycone.

Noteworthy is that several matrices can be used in the same platform to produce the purified fractions having different polyphenols compositions with a high added value. For example, from olive leaves, the final products are OLP1 and OLP2 powders containing a high level of polyphenols and of OLE. OLP1 and OLP2 powders, produced from Frantoio and Leccino leaves, using membrane purifications and vacuum concentrations, show different polyphenol concentration whose main component is OLE, as reported in Table 5. As a representative HPLC-DAD profile of a sample, Figure 2 reports OLP1 at 280, 240, and 330 nm.

**Table 5:** Polyphenolic composition of olive leaves powders OLP1 and OLP2 after extraction, membrane purification, under vacuum concentration, and final dryer procedure.

**Compound(s)**	**OLP1**	**OLP 2**
	**mg/g**	**mg/g**
Hydroxytyrosol glucoside	2.6 ± 0.1	1.31 ± 0.05
Hydroxytyrosol	1.02 ± 0.04	nd
Demethyl elenolic acid diglucoside	(1.53 ± 0.06) × 10	(1.03 ± 0.04) × 10
Caffeic acid derivative	2.21 ± 0.09	nd
Verbascoside	6.3 ± 0.2	3.0 ± 0.1
Luteolin 7-O-glucoside	3.6 ± 0.1	1.84 ± 0.07
Oleuropein	(3.5 ± 0.2) × 10^2^	(2.40 ± 0.09) × 10^2^
Oleuropein aglycone	(2.9 ± 0.1) × 10	2.5 ± 0.1
*Total polyphenols*	(4.1 ± 0.2) × 10^2^	(2.6 ± 0.1) × 10^2^

*nd, not detected*.

As a final example, an industrial extract enriched in secoiridoids, in particular OLC and oleacin (OLEA), the dialdehydic form of elenolic acid conjugated with 3,4-(dihydroxyphenyl) ethanol, was obtained from LC separation after EVOO hydroalcoholic ultrasound extraction and treatment. The chemical composition is reported in Table 6.

**Table 6:** Polyphenolic composition of an EVOO industrial extract enriched in secoiridoids.

**Compound(s)**	**mg/g**
Hydroxytyrosol	(1.24 ± 0.05) × 10
Tyrosol	(8.3 ± 0.3)
Elenolic acid	(7.6 ± 0.3) × 10
10-Hydroxyoleocanthal	(2.9 ± 0.1) × 10^2^
Oleocanthal	(1.89 ± 0.07) × 10^2^
Oleuropein aglycone	(9.5 ± 0.4) × 10
Ligstroside aglycone	(2.33 ± 0.09) × 10
Ligustaloside A + B	(1.72 ± 0.07) × 10^2^
*Total polyphenols*	(8.7 ± 0.3) × 10^2^

Noteworthy is that the continuous interaction between high technology and environmental and economic sustainability makes this multifunctional platform highly innovative and consistent with the circular economy strategy. In fact, wastes are used as starting materials, and each residue of the process (water, olive stones, and destoned pulp) would come into new use in the same and/or further processed according to the “zero waste concept.”

#### Biological Activities

As previously shown by a large number of studies with special regard to CDNCDs, among the polyphenols present into EVOO, one that has beneficial effects on human health is OLC (31, 53, 54).

OLC exhibits anticancer activity (55) through different mechanisms of action. In fact, the proliferation of neoplastic cells can be controlled by phosphorylation of tyrosine-protein kinase Met (c-Met). Studies *in vitro* have shown that OLC induces a reduction of c-Met receptor expression, which seems to be involved in the angiogenesis processes and the growth of the tumor mass [Table 7; (60)].

**Table 7:** Principal studies on extra-virgin olive oil biological effects.

**Type of the study**	**References**	**Outcome**	**Conclusions**
*In vitro*	(33)	Evaluation of the effects of MPC on NF-kβ in monocyte and monocyte-derived macrophages sampled from healthy subjects.	MPC of EVOO inhibits the translocation of p50 and p65 of NF-kβ in both cell types.
	(56)	Evaluation of the HT activity on human colorectal adenocarcinoma cell lines.	HT seems to arrest the cell cycle in the G2/M phase, demonstrating an anti-tumoral activity.
	(57)	OLC inhibits the proliferation, migration, and invasion of epithelial human breast and prostate cancer cellular lines.	Results confirm the therapeutic role of OLC in c-Met depended on cancers.
	(58)	*p*-HPEA-EDA inhibits cellular transformation in JB6CL41 cell lines and suppresses COX-2 and its carcinogenicity in HT-29 colon cancer cells.	*p*-HPEA-EDA would like to exert a chemopreventive role against colon cancer cells.
	(59)	Evaluation of the possible anticancer effect of HT in human colon cancer cells.	HT induces apoptotic cell death and mitochondrial dysfunction through ROS production in colon cancer cells.
	(60)	Assessment of the anticancer effects of OLC related to the c-Met receptor.	OLC can control malignancies with aberrant c-Met activity.
	(61)	Evaluation of the possible effects of OLEA (10 and 20 μmol/l) on the expression of IL-10 and CD63 receptor.	OLEA increases the anti-inflammatory activity of Hb-haptoglobin complexes.
	(62)	OLC inhibits colonies formation and induces apoptosis in hepatocellular and colorectal carcinoma cells.	OLC increases intracellular ROS production and causes the mitochondrial depolarization, inducing an anticarcinogenic action against hepatocellular carcinoma e and colorectal carcinoma cells.
	(63)	Evaluation of OLE from olive leaves on A375 human melanoma cells.	OLE alone or in combination with chemotherapeutics can stimulate apoptosis in human melanoma cells.
Animal	(64)	Evaluation of EVOO effects on platelet aggregation and homocysteine plasma concentration to MPC components.	MPC of EVOO inhibits the platelet aggregation and reduce the homocysteine plasma concentration with CV protection.
	(65)	Assessment of the possible effects in reducing lipid profile and increase antioxidant capacity with triacetylated (t) HT vs. HT purified, in rats.	The assumption both of triacetylated (t)HT vs. HT purified showed a hypolipemic effect and an increase of antioxidant capacities.
	(66)	Evaluation of the antiplatelet effects of HT and HT-AC compared to ASA.	The administration of HT and HT-AC inhibits platelet aggregation and reduces thromboxane synthesis through enhanced production of NO.
	(67)	Assessment of OLEA protective effects on the damage and metabolic alterations induced by HDF.	HDF related to hepatic insulin resistance could be partly prevented by oral supplementation of OLEA.
	(68)	Evaluation of the possible effects of HT on inflammatory markers such as COX-2 and TNF-α in mouse models of systemic inflammation.	HT prevents LPS induced effects and ameliorates the antioxidant capacity of plasma.
	(69)	Assessment of the neuroprotective effects of HT.	HT treatment seems to counteract the decline of neuro-angiogenesis related to aging.
Human	(70)	Examination of the influence of 40 ml of EVOO weekly on maximum platelet aggregation.	Platelet aggregation is influenced by EVOO acute intake and by EVOO phenolic composition.
	(71)	Evaluation of atherosclerotic plaque from 20 hypertensive subjects, to evaluate the effects of OLEA on Hb-haptoglobin complex and its effects on change macrophage phenotype from pro-inflammatory M1 to anti-inflammatory M2.	OLEA could mitigate the destabilization of carotid plaque, reducing the ischemic stroke risk.

*ASA, acetylsalicylic acid; c-Met, tyrosine-protein kinase Met; COX-2, cyclooxygenase- 2; CV, cardiovascular; Hb, hemoglobin; HDF, high-fat diet; HT, hydroxytyrosol; HT-AC, hydroxytyrosol acetate; LPS, lipopolysaccharide; MPC, Minor polar compounds; NF-kβ, nuclear factor kappa-light-chain-enhancer of activated B cells; NO, nitric oxide; OLC, oleocanthal; OLE, oleuropein; OLEA, oleacin; p-HPEA-EDA, decarboxymethyl ligstroside aglycone; ROS, reactive oxygen species; t(HT), triacetylated hydroxytyrosol; TNF-α, tumor necrosis factor-α*.

Elnagar et al. (57) demonstrated the anti-angiogenic activity of OLC in cell lines of human breast and prostate cancer (MCF7, MDA-MB-231, and PC-3) through the downregulation of CD31 expression (a biomarker of the microvessel density).

Decarboxymethyl ligstroside aglycone (p-HPEA-EDA) inhibits COX-2 and activates AMPK in HT-29 colon cancer cells in “*in vitro*” model (58), and OLC would seem to promote apoptosis, which in turn eradicates tumor or counteracts its growth. Specifically, OLC increases the phosphorylation of extracellular signal-regulated kinase (ERK1/2), inducing cell death rapidly. Moreover, it leads to a change in the permeability of the lysosomal membrane, favoring the release of pro-apoptotic enzymes in tumor cells (72). OLC has been shown to perform an incisive antiproliferative action and a downregulation of the mechanistic target of rapamycin in breast cancer cell lines (73).

Cusimano et al. (62) showed that OLC can increase the levels of reactive oxygen species (ROS) in colon and liver cancer cells, caused increased cell death.

Several studies demonstrated that a constant low dose of aspirin (another COX inhibitor) is cardioprotective (39). Therefore, it can be speculated that the long-term consumption of OLC can have a cardioprotective role. Currently, OLC cardioprotective action has been little investigated; in fact, a single study highlights its possible protective effects in atherosclerotic CV disease (74). This pathological condition is a chronic inflammatory process that involves the vessel walls, starting from the endothelium. The damage to the endothelial is mainly caused by platelet aggregation (74, 75). Recently, Agrawal et al. (70) have shown, in a randomized clinical trial, that the intake of 40 ml weekly of EVOO rich in OLC can influence platelet aggregation in healthy male adults, confirming previous data obtained from animal studies (64).

Moreover, an *in vitro* study demonstrated that polyphenolic extracts of EVOO inhibit nuclear factor kβ (NF-kβ) in monocytes and monocytes derived macrophages sampled from healthy subjects (33). This effect was particularly evident on the p50 subunit of NF-kβ, leading to a reduced expression of vascular cell adhesion molecules-1 and a decreased adherence of leukocytes to the endothelium, promoting normal endothelial function (76).

Another of the most abundant phenolic compounds of EVOO is OLEA. Although numerous beneficial health effects are ascribed to OLEA, its main characteristic would seem to be the attenuation of the destabilization of the carotid plaque (71, 77); in fact, its use seems to be effective in the reduction of ischemic stroke risk.

As demonstrated in an *ex vivo* study by Filipek et al. (71), the OLE mechanism of action would be aimed at reducing the release of high mobility group protein-1 (up to 90%), matrix metalloproteinase (MMP)-9 (up to 80%), complex MMP-9 and neutrophil gelatinase-associated lipocalin complex, as MMP-9/neutrophil gelatinase-associated lipocalin complex (up to 80%) and tissue factor (TF) (over 90%) in treated carotid plaques compared with those of the control group.

Further action of OLEA is a scavenger of free radicals: its remarkable antioxidant action is due to the decrease in myeloperoxidase (MPO) release by human neutrophils. MPO is an enzyme able to catalyze the formation of pro-oxidant species, highly reactive to oxygen (such as HClO) and chlorination reactions (78). OLEA can significantly reduce the release of MPO from neutrophils in a comparable manner with the nonsteroidal anti-inflammatory drug (77).

However, in addition to the antioxidant action, OLEA shows anti-inflammatory effects through the stimulation of macrophage activity (increase in the expression of CD163 macrophage receptors) (61). It also decreases the adhesion of monocytes to vascular endothelial cells by inhibiting the expression of adhesion molecules E-selectin, vascular cell adhesion molecules-1, and intercellular adhesion molecule-1 (77). OLE also displays a cardioprotective action by inhibiting the angiotensin-converting enzyme, and animal models suggest that OLEA irreversibly inhibits angiotensin-converting enzyme through a tight bond (79, 80). Additional positive actions induced by OLEA on health were also described in a study by Lombardo et al. (67). Beyond the already known anti-inflammatory and antioxidant actions described in the literature (77, 81), its potential actions would concern metabolic alterations induced by a high-fat diet (HDF) in an animal study. The study was divided into two phases: the first phase included the administration of an HDF with daily oral supplementation of OLEA, whereas the second phase provided the administration of OLEA in already obese animal models. In the first phase, these authors have shown that a daily intake of OLEA (20 mg/kg) for 5 weeks can exert a protective role on several metabolic alterations related to HDF. In particular, statistically significant improvements were observed in the reduction of body weight, the latter due to the decrease in visceral abdominal adipose tissue and the reduction of both microscopic and macroscopic hepatic steatoses. Moreover, other findings were referred to lowering of serum lipids and lower postprandial glycemic values, which prove greater insulin sensitivity. In the second phase, OLEA has only partially shown some beneficial effects vs. HDF. In fact, a reduced weight and lipid infarction of the liver was observed, but no positive effects on total cholesterol glycemia or triglyceride levels were shown. These data confirm the thesis that OLEA can induce beneficial effects in the prevention in dysmetabolic conditions such as weight gain and insulin resistance, but it cannot show the same protective effects when dysmetabolic conditions are already established.

HT is phenolic alcohol that is present in EVOO mainly derived from hydrolysis of OLE (56, 82) exhibiting a wide plethora of biological effects (83, 84) as cardioprotective, anticancer, antimicrobial, neuroprotective, and endocrine, although its molecular mechanism has not yet been clarified (85–87). Despite its content is known in EVOO, starting from 2006 (specifically with the European Regulation 1924/2006) (88, 89), it is allowed to report health claims in labeling, presentation, and advertising. In this context, the European Food Safety Authority has authorized the health claim for EVOO according to their HT and its derivatives content, which must be at least 5 mg per 20 ml of EVOO (90).

HT is one of the most powerful natural antioxidant extracts, just after gallic acid (91). The main feature of HT is to act as an antioxidant compound through the activity of free radical scavenger and metal chelator (85). In addition to the well-known biochemical properties, HT would seem to be able to provide protective actions against oxidative stress by activating different cellular pathways, increasing the endogenous defense system (68). The mechanism underlying this action would lie in the ability of HT to interact with phase II detoxification enzymes through the activation of the nuclear factor erythroid 2-related factor 2 in different tissues (92, 93).

One of the main features of HT is to perform a cardioprotective action. The main pathogenetic mechanism underlying the onset of CV disease is the endothelial dysfunction caused by an increase in ROS and dyslipidemia. In this context, HT showed remarkable capacities in preventing lipid peroxidation and counteracting the oxidation of low-density lipoprotein cholesterol (94). In an animal model study, Jemai et al. (65) tested triacetylated (t)HT vs. HT (purified from olive tree leaves) to evaluate lipid-lowering and antioxidative activity. Two arms of Wistar rats fed for 16 weeks with standard laboratory diet vs. diet with high-cholesterol content were studied for serum lipid values, lipid peroxidation (the latter assessed by thiobarbituric acid reactive substances), superoxide dismutase levels, and catalase (CAT) levels. The group fed with high-cholesterol diet showed an increase in total cholesterol, triglycerides, and low-density lipoproteins, but after administration of tHT and HT (3 mg/kg of body weight), total cholesterol, triglycerides, and low-density lipoproteins ameliorate in a statistically significant manner. Also, higher HDL cholesterol levels were present after the oral administration with tHT and HT. This study also investigated the lipid peroxidation levels that showed lower values in the group administered HT and its triacetylated derivatives compared with the group fed with the hypercholesterolemic diet. The activity of superoxide dismutase and CAT in the liver was also found to be higher. The results obtained by this study show the potential beneficial action of HT and its triacetylated derivatives on the lipid profile and lipid peroxidation, improving the cellular antioxidant capacity.

A further cardioprotective role carried out by HT and its derivatives is the ability to decrease thrombogenic processes through the regulation of platelet aggregation. This protective effect on platelet aggregation was showed in an animal study by Gonzalez-Correa et al. (66) through the comparison of the inhibition of platelet synthesis between HT, HT acetate, and acetylsalicylic acid.

In recent years, numerous studies have investigated potential HT antitumor activity (87, 95, 96), and they focused on the correlation between HT and colorectal cancer. Stoneham et al. (97) found an association between secondary bile salts pattern and HT. In fact, secondary bile salts inhibit diamine oxidase (an enzyme that catabolizes histamines), which in turn could promote the progression of colonocytes from mucosa to carcinoma and/or adenocarcinoma. HT may have a protective role against this condition, influencing secondary colon bile patterns slowing the progression of normal mucosa to carcinoma and/or adenocarcinoma.

Further studies have shown that HT decreases the expression of B-cell lymphoma-2 and COX-2 (98).

HT can reduce DNA damage in human colon cancer HT29 cells (56, 99). Gill et al. (99) tested HT *in vitro* on HT29 cells, at concentrations of 0, 5, 10, 25, 50, 75, and 100 μg/ml pretreated for at least 24 h. The results obtained showed the inhibition of cellular invasion (concentrations of 25, 50, 75, and 100 μg/ml) and cell adhesion (concentrations of 75 and 100 μg/ml). No significant effect was identified in the expression process cell related to metastasis (99).

In DLD1 cell lines of human colon cancer, Sun et al. (59) showed that HT could induce oxidative stress preferentially on colon cancer cells rather than normal colon epithelial cells. HT can activate phosphoinositide 3-kinase/Akt pathway, forkhead box O3 (FOXO3) phosphorylated, and therefore downregulated FOXO3 target genes. The results obtained showed that HT induces cell death and endothelial dysfunction by generating ROS (such as superoxide anion and H_2_O_2_) in colon cancer cells.

In addition to effects on colon cancer, HT has also been investigated in other types of cancer, such as breast, hepatic, skin, and others (100, 101).

A recent *in vivo* study evidenced that HT stimulates neurogenesis in aged dentate gyrus by enhancing stem and progenitor cell proliferation and neuron survival (69).

Olive leaf extract and its main component OLE prevent chronic ultraviolet B radiation-induced skin damage and carcinogenesis in hairless mice (102). Moreover, in a previous study conducted by our group, we observed that OLE extract from olive leaves, alone or in combination with chemotherapeutics, exerts a cytotoxic action on A375 human melanoma cells (63). We showed that OLE was able, at a dose of 500 μM, to stimulate apoptosis in melanoma cells. Therefore, OLE could represent a new therapeutic approach, which could support chemotherapy therapy.

### Micronized Powders and Extracts Enriched in Polyphenols From *Vitis vinifera* L. Wastes

#### Production and Chemical Characterization

**Figure 3:**
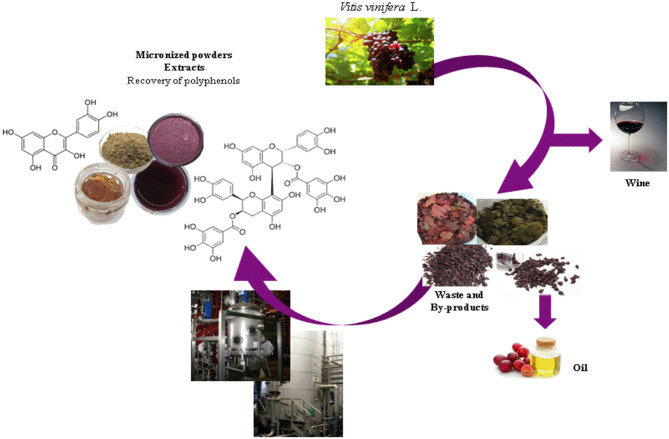
Winemaking process and valorization of Vitis wastes.

According to the circular economy strategy, winery wastes can be used to obtain industrial micronized powders and extracts that can be used for agronomic, food, nutraceutical, cosmetic, and pharmaceutical applications generating a parallel market to that of the wine production (Figure 3). Grape seeds represent the most precious winery wastes in terms of concentration of polyphenols (103–105) showing a wide number of biological activities (106, 107). Also, after the removal of polyphenols, grape seeds can be used as biomass for the generation of energy (37).

**Figure 4:**
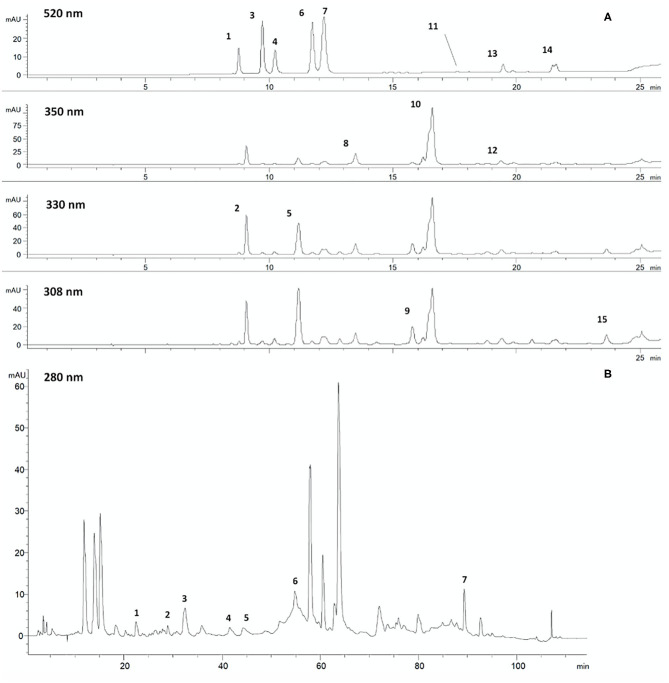
Chromatographic profiles of a hydroalcoholic extract from dried grape skins: **(A)** 520, 350, 330, and 308 nm, chromatographic method for anthocyanosides, flavonols, hydroxycinnamic derivatives, phenolic acids, stilbenes. 1. Delphinidin 3-O-glucoside; 2. Caftaric acid; 3. Cyanidin 3-O-glucoside; 4. Petunidin 3-O-glucoside; 5. Coutaric acid; 6. Peonidin 3-O-glucoside; 7. Malvidin 3-O-glucoside; 8. Quercetin 3-O-glucuronide; 9. *trans*-Resveratrol glucoside; 10. Quercetin 3-O-glucoside; 11. Malvidin 3-O-acetyl glucoside; 12. Kaempferol 3-O-glucoside; 13. Malvidin 3-O-caffeoyl glucoside; 14. Malvidin 3-O-coumaroyl glucoside; 15. *cis*-Resveratrol. **(B)** 280 nm, chromatographic method for proanthocyanidins. 1. Catechin dimer B3; 2. Catechin; 3. Catechin dimer B6; 4. Epicatechin; 5. Catechin trimer; 6. Catechin oligomers; 7. Catechin/epicatechin trimers digallated.

As shown in Table 2, dried Vitis wastes consist of red and green leaves, grape marcs, grape skins, and grape seeds after wine production. By drying and micronization processes, functional powders and commercial extracts enriched in different polyphenol subclasses are produced for food and cosmetic uses. The following are the HPLC-DAD-MS characterizations of individual polyphenolic compounds found in some representative powders and extracts from leaf and grape seeds before and after the production of oil. Figure 4 shows an example of the chromatographic profile of a hydroalcoholic extract from PSAG-DP, grape peels (Sagrantino).

Table 8 reports the polyphenol content of the dried fractions of several samples: PSAN, dried grape marcs (Sangiovese); PSAG, dried grape marcs (Sagrantino); PSAG-DP, grape peels (Sagrantino), dry powder; SSAG-DP, grape seeds after oil extraction, dry powder; and LIOP-PH, lyophilized grape marcs. Table 8 also shows the polyphenolic content of SGR-C, a commercial grape seeds powder obtained by hydroalcoholic extraction, purification through membrane technologies, concentration from vacuum evaporation, and dehydration.

**Table 8:** Characterization of grape marcs and derived products.

**Compound(s)**	**PSAN**	**PSAG**	**PSAG-DP**	**SSAG-DP**	**SGR-C**	**LIOP-PH**
	**mg/g dried vegetal material**					
Delphinidin-3-glucoside	0.0062 ± 0.0003	0.086 ± 0.005	0.040 ± 0.002	nd	nd	0.028 ± 0.001
Cyanidin-3-glucoside	0.0180 ± 0.0006	0.0172 ± 0.0006	0.079 ± 0.003	nd	nd	0.0021 ± 0.0001
Petunidin-3-glucoside	0.0202 ± 0.0007	0.101 ± 0.002	0.064 ± 0.002	nd	nd	0.038 ± 0.002
Peonidin-3-glucoside	0.186 ± 0.002	0.041 ± 0.002	0.265 ± 0.006	nd	nd	0.0173 ± 0.0007
Malvidin-3-glucoside	0.171 ± 0.003	0.36 ± 0.01	0.249 ± 0.005	nd	nd	0.224 ± 0.008
Cyanidin-3-acetyl glucoside	nd	0.022 ± 0.001	0.0073 ± 0.0004	nd	nd	nd
Peonidin-3-acetyl glucoside	0.189 ± 0.002	nd	nd	nd	nd	nd
Malvidin-3-acetyl glucoside	0.234 ± 0.006	0.057 ± 0.002	0.078 ± 0.004	nd	nd	nd
Malvidin-3-caffeoyl glucoside	nd	nd	0.0124 ± 0.0005	nd	nd	nd
Petunidin-3-cumaroyl glucoside	nd	nd	0.0160 ± 0.0006	nd	nd	nd
Peonidin-3-coumaroyl glucoside	nd	nd	0.022 ± 0.001	nd	nd	nd
Malvidin-3-coumaroyl glucoside	nd	0.380 ± 0.008	0.098 ± 0.004	nd	nd	0.195 ± 0.008
Other anthocyanins (expressed as malvidin-3-glucoside)	0.206 ± 0.004	0.270 ± 0.008	nd	nd	nd	0.188 ± 0.005
Delphinidin aglycone	1.13 ± 0.01	nd	0.287 ± 0.005	nd	nd	nd
Gallic acid	0.032 ± 0.002	0.077 ± 0.001	0.0191 ± 0.0008	0.036 ± 0.002	0.0039 ± 0.0002	0.142 ± 0.004
Catechin dimer B3	0.68 ± 0.03	0.152 ± 0.008	nd	1.51 ± 0.02	2.22 ± 0.07	0.33 ± 0.01
Catechin	0.199 ± 0.004	0.082 ± 0.003	0.0303 ± 0.0009	1.0 ± 0.2	(1.10 ± 0.02) x 10	nd
Catechin trimer	0.39 ± 0.02	nd	nd	0.434 ± 0.009	3.21 ± 0.06	1.03 ± 0.03
Catechin dimer B6	nd	nd	nd	0.66 ± 0.01	2.61 ± 0.06	0.32 ± 0.01
Catechin dimer B2	0.212 ± 0.009	0.42 ± 0.01	nd	0.78 ± 0.02	5.37 ± 0.09	0.19 ± 0.01
Epicatechin	0.234 ± 0.008	nd	0.032 ± 0.001	0.88 ± 0.03	(1.36 ± 0.03) x 10	0.073 ± 0.002
Catechin trimer	nd	0.216 ± 0.006	nd	0.40 ± 0.01	3.7 ± 0.1	nd
ECG dimers	nd	nd	0.89 ± 0.02	1.47 ± 0.06	6.6 ± 0.2	nd
Epicatechin gallate	nd	nd	nd	nd	6.1 ± 0.2	nd
Catechin oligomers expressed as tetramers	1.80 ± 0.02	0.97 ± 0.02	nd	(1.70 ± 0.02) × 10	(5.49 ± 0.08) × 10	2.46 ± 0.05
ECG dimers	nd	3.00 ± 0.03	nd	(1.27 ± 0.04) × 10	(1.81 ± 0.03) × 10^2^	5.09 ± 0.09
Catechin/epicatechin trimers digallated	8.68 ± 0.09	(1.21 ± 0.01) × 10	(1.07 ± 0.01) × 10	(3.18 ± 0.06) × 10	(5.3 ± 0.1) × 10^2^	(1.48 ± 0.02) × 10
Flavonols expressed as kaempferol 3-O-glucoside	0.138 ± 0.005	nd	nd	nd	nd	nd
*Total polyphenols*	(1.46 ± 0.02) × 10	(1.83 ± 0.02) × 10	(1.29 ± 0.02) × 10	(6.9 ± 0.2) × 10	(8.2 ± 0.2) × 10^2^	(2.51 ± 0.04) × 10

*PSAN, Dried grape marcs (Sangiovese); PSAG, Dried grape marcs (Sagrantino); PSAG-DP, grape peels (Sagrantino), dry powder; SSAG-DP, grape seeds after oil extraction, dry powder; SGR-C, commercial grape seeds extract; LIOP-PH, lyophilized grape marcs; nd, no detected*.

Dried grape leaves and seeds after grape seed oil productions are used for hot aqueous or hydro-alcoholic extraction of standardized fractions in polyphenolic compounds (Tables 9–11). A similar extraction from green or red Vitis leaves allows us to obtain commercial powder extracts with content of flavonoids derived from quercetin, kaempferol, and hydroxycinnamate acids ranging from 4 to 25%. The content in catechins and procyanidins is low, but the extracts of red vine contain up to 3% of anthocyanosides. They can be applied in the cosmetic and food supplements sectors.

**Table 9:** Characterization of grape leaves of different cultivars.

**Compound(s)**	**SAG**	**SAN**	**MER**
	**mg/g green leaves**		
Caffeoyl-tartaric acid	1.18 ± 0.02	0.33 ± 0.01	0.437 ± 0.009
Caffeic acid derivatives expressed as chlorogenic acid	0.081 ± 0.004	0.0051 ± 0.0002	0.0104 ± 0.0004
Rutin	2.13 ± 0.04	1.07 ± 0.04	1.41 ± 0.03
Flavonols expressed as rutin	0.49 ± 0.02	0.29 ± 0.01	0.83 ± 0.03
*Total polyphenols*	3.88 ± 0.08	1.69 ± 0.06	2.69 ± 0.07

**Table 10:** Grape leaves commercial extract from extraction, membrane purifications, and vacuum evaporation.

**Compound**	**mg/g**
Caffeoyl-tartaric acid	0.56 ± 0.02
*p*-Coumaroyl-tartaric acid	0.067 ± 0.003
Feruloyl-tartaric acid	0.103 ± 0.003
Quercetin 3-O-glucuronide	0.93 ± 0.02
Quercetin 3-O-rutinoside	0.132 ± 0.005
Quercetin 3-O-galattoside	0.0052 ± 0.0003
Quercetin 3-O-glucoside	0.106 ± 0.002
*Total polyphenols*	1.90 ± 0.05

**Table 11:** Phenolic composition of grape seed extract.

**Compound(s)**	**mg/g**
Gallic acid	0.042 ± 0.002
Catechin dimer B3	1.69 ± 0.03
Catechin	0.82 ± 0.02
Catechin trimer	nd
Catechin dimer B6	1.29 ± 0.03
Catechin dimer B2	0.78 ± 0.03
Epicatechin	0.58 ± 0.02
Catechin trimer	0.49 ± 0.02
ECG dimers	1.65 ± 0.03
Catechin oligomers quantified as tetramers	(2.62 ± 0.05) × 10
ECG dimers	(1.71 ± 0.04) × 10
Catechin/epicatechin trimers digallated	(3.94 ± 0.08) × 10
Catechin/epicatechin trimers digallated	4.5 ± 0.1
*Total polyphenols*	(9.5 ± 0.2) × 10

*nd, not detected*.

The polyphenolic content of grape seed extract (GSE) is reported in Table 12. As shown, the main compounds are catechin and epicatechin monomers, dimers, and trimers.

**Table 12:** Extract from grape seed after oil extraction.

**Compound(s)**	**mg/g**
Gallic acid	0.41 ± 0.02
Catechin dimer B3	1.52 ± 0.04
Catechin	0.64 ± 0.01
Catechin trimer	0.54 ± 0.02
Catechin dimer B6	0.77 ± 0.03
Catechin dimer B2	0.96 ± 0.04
Epicatechin	0.59 ± 0.02
Catechin trimer	nd
ECG dimers	1.06 ± 0.02
Catechin oligomers quantified as tetramers	9.5 ± 0.2
ECG dimers	5.5 ± 0.2
Catechin/epicatechin trimers digallated	(1.35 ± 0.04) × 10
Catechin/epicatechin trimers digallated	8.9 ± 0.2
*Total polyphenols*	(4.4 ± 0.1) × 10

*nd, not detected*.

#### Biological Activities

In recent years, scientific interest has shifted to the recovery of its waste components to obtain products that are able to perform beneficial actions on human health. In this context, *Vitis vinifera* L. grape seed, grapevine leaves, and grape pomace are currently widely studied for their potential health properties (Table 13).

**Table 13:** Principal studies on *Vitis vinifera* L. biological effects.

**Type of the study**	**References**	**Outcome**	**Conclusions**
Animal	(108)	Evaluation of the possible effects of GSE on hepatic and renal dysfunction induced by dexamethasone.	Treatment with GSE induces an increase in the GSH and catalase activity.
	(109)	Evaluation of acute and subacute hypoglycemic effects of the aqueous extracts from *Vitis vinifera* L. leaves in induced diabetic rats.	Extracts from *Vitis vinifera* L. leaves exert a significant antihyperglycemic and antioxidant activity.
	(110)	Hepatoprotective effects of ethanolic extracts and its four different fractions of *Vitis vinifera* L. in induced acute liver damage.	Ethanolic extracts showed an antioxidant and hepatoprotective activity assessed by AST, ALT, and GSH levels.
	(111)	Evaluation of the protective effects of GSE against oxidative liver injury and fibrosis caused by biliary obstruction.	GSE protects oxidative liver damage induced by bile duct obstruction, through inhibition of neutrophil infiltration and lipid peroxidation.
	(112)	Evaluation of antioxidant and anti-hypercholesterolemic action of the aqueous extracts from *Vitis vinifera* L. leaves.	Natural active compounds such as tannins, flavonoids and phenolic acids exert a significative anti-hypercholesterolemic and antioxidant action.
	(113)	Evaluation of grape pomace possible effects on blood pressure values.	Grape pomace induces hypertensive action on systolic blood pressure values, in spontaneously hypertensive rats. When the treatment was suspended, the authors observed a “rebound” effect on systolic blood pressure values.

*ALT, alanine aminotransferase; AST, aspartate transaminase; GSE, Grape seed extracts; GSH, glutathione*.

The biological properties of the seeds of *Vitis vinifera* L. are due to their high content in polyphenols, including flavonoids (proanthocyanidins), which have an antioxidant, anti-inflammatory and also cardioprotective, hepatoprotective, hypoglycemic action and are neuroprotective (114).

Several studies reveal that GSE inhibits the enzymes responsible for the radicals formation and it has anticancer effects (115, 116).

An animal study evaluated the potential effects of GSE on oxidative liver injury and fibrosis caused by biliary duct obstruction (BDL). The author divided the rats into four subgroups: the control group, the group treated with oral administration of GSE (at a dose of 50 mg/kg/day for 28 days), the group with bile duct ligated, and finally, the group treated with oral administration of GSE having bile duct ligated. They found that aspartate transaminase, alanine aminotransferase, lactate dehydrogenase, and tumor necrosis factor-α were increased in BDL concerning the control group. Inversely, the same parameters were significantly reduced in the GSE group compared with those in the control group. Total antioxidant capacity and liver levels of glutathione (GSH) were reduced in the BDL group and returned to normal levels in the BDL group treated with GSE. Therefore, GSE would seem to protect the liver from oxidative damage induced by the obstruction of the bile duct in rats, as it inhibits the infiltration of neutrophils and lipid peroxidation, restoring a balance between the antioxidant and oxidant capacity of the liver (111). The following animal study assessed the protective of GSE against kidney and liver dysfunction caused by dexamethasone in female albino rats. The authors divided the rat population into four subgroups: group 1 (control group), group 2 (subcutaneous injection with dexamethasone at a dose of 0.1 μg/kg/body weight), group 3 (subcutaneous injection with dexamethasone at a dose of 0.1 μg/kg/body weight and oral assumption of GSE at a dose of 200 mg/kg/body weight), and group 4 (subcutaneous injection with dexamethasone at a dose of 0.1 μg/kg/body weight and oral assumption of GSE at a dose of 400 mg/kg/body weight). After 4 weeks of dexamethasone treatment, group 2 showed an increase of liver and renal function biomarkers (such as aspartate transaminase, alanine aminotransferase, uric acid, creatinine, and glucose) and a reduction in liver GSH, CAT, and glucose-6-phosphate dehydrogenase. The group treated with GSE showed an increase in liver GSH and CAT, demonstrating a positive effect of GSE against hepatic and renal damage induced by glucocorticoids (108).

An animal study assessed the possible antidiabetic (hypoglycemic) activities of the aqueous extracts of *Vitis vinifera* leaves in normoglycemic, hyperglycemic rats and induced diabetic rats. The authors assessed the effects induced by 15-day assumption of aqueous extracts from *Vitis vinifera* L. leaf at two different doses (250 and 500 mg/kg body), and they showed that these extracts had a significant antioxidant and hypoglycemic action similar to that of tolbutamide, the reference hypoglycemic substance (109).

A subsequent animal study also highlighted the hepatoprotective effect of Vitis leaves, as it had been shown for the seeds. The authors studied these beneficial effects on the liver in acute liver injury mouse models. The pretreatment with *Vitis vinifera* L. leaf extracts reduces the increase in liver damage markers. Therefore, these fractions increase the function of the hepatocytes, both by biochemical markers and histological findings. The hepatoprotective effect is due to the inhibition of cytochrome P450 with consequent prevention of lipid peroxidation and stabilization of the membrane of the hepatocyte (110).

A recent animal study has also shown the cholesterol-lowering effect of *Vitis vinifera* L. red leaf extract due to its active phytoconstituents, among which resveratrol plays a fundamental role for its anti-atherosclerotic and anti-dyslipidemic properties (112).

An animal study has shown that after the winemaking process, polyphenol levels in the grape pomace are in sufficient quantity to exert an antihypertensive action. Furthermore, the composition of the extracts used would seem to modulate the antihypertensive effect by reducing or increasing the amount of polyphenols present (113).

## Conclusions

Polyphenols are natural compounds found in plant foods exhibiting a variety of beneficial effects on human health. For this reason, the global polyphenol market is continuously on the rise. In response to this growing demand, agro-industrial wastes could offer a solution.

This paper reported the high potential of wastes deriving from olive oil and wine production sectors. In particular, an industrial platform for the production of micronized powders and extracts from *Olea europaea* L. and *Vitis vinifera* L. wastes using advanced drying techniques and membrane technologies, and, when necessary, evaporation was described. This platform is environmentally sustainable; in fact, the final products were obtained using water as a solvent of extraction, avoiding the toxic solvents. Powders and extracts are standardized in terms of polyphenol content, as confirmed by the HPLC-DAD-MS analysis. The platform is also economically sustainable because it can be used all year, processing the agro-industrial wastes of different seasons.

To the best of our knowledge, this is the first work that brings back for Olea the innovative production of oil-free olive paste powders, standardized in HT content for food, feed, and cosmetics. Another innovation of this paper concerns the production and characterization of bioactive commercial products enriched in OLC and OLEA extracted from EVOO. As for Vitis wastes, they have been proposed for the first time for the production of grape peel powder rich in anthocyanins and a commercial product obtained from wine waste, enriched in quercetin and its derivatives.

Due to their high content of polyphenols, responsible for many biological properties, micronized powders and extracts are promising for biomedical applications, assisting clinicians in contrasting the onset and progression of CDNCDs. In particular, data not reported in this work, for the different fractions enriched and micronized by both Olea and Vitis, the research and development activities of *in vitro* and *in vivo* tests are reported later.

In a previous study conducted by our group, we found that an olive leaf extract enriched in OLE, named OLEO, decreased melanoma cell proliferation and motility and was also able to reduce the rate of glycolysis of human melanoma cells without affecting oxidative phosphorylation. OLEO represents a natural product effective in reducing the glycolytic metabolism of different tumor types, revealing an extended metabolic inhibitory activity that may be well suited in a complementary anticancer therapy (63).

A similar study is in progress on an extract enriched in HT and human melanoma cells. Also, an *in vitro* study is underway on the anti-inflammatory activity of the EVOO fraction on murine macrophages. In this view, our research group is conducting an *in vivo* study in patients affected by chronic kidney disease to evaluate the possible health effects induced by different EVOOs, naturally enriched in polyphenols and secoiridoids. This study, named EXTRANUTRAOILS, has been approved by the Ethical Committee of the University Hospital Tor Vergata of Rome (protocol number: 36.20). As regards micronized fractions from Vitis, the *in vitro* tests are underway on human kidney cells to verify antioxidant and detoxifying properties as a preliminary phase of an *in vivo* study, the W.I.S.E. study. This study assesses the impact of the red wine components in improving physical and psychological outcomes in uro-oncological patients. The W.I.S.E. is a randomized, controlled dietary intervention trial in progress and carried out at the Urological Research Institute of San Raffaele Hospital.

## Data Availability Statement

The raw data supporting the conclusions of this article will be made available by the authors, without undue reservation.

## Author Contributions

AR, RB, and AN have projected the study and have written the paper. MC, SU, and GM have performed the experiments. All authors revised the final version of the paper.

## Conflict of Interest

The authors declare that the research was conducted in the absence of any commercial or financial relationships that could be construed as a potential conflict of interest.

## Abbreviations

AD: after AP drying
AMPK: activated protein kinase
AP: after oil pressing
B-Raf: serine/threonine-protein kinase
BBC: basal cell carcinoma
BDL: biliary duct obstruction
c-Met: tyrosine-protein kinase
CAT: catalase
CDNCDs: chronic degenerative non-communicable diseases
COX: cyclooxygenases
cSCC: cutaneous squamous cell carcinoma
CV: cardiovascular
ERK: extracellular-signal regulated kinase
EVOO: extra-virgin olive oil
FOXO3: forkhead box O3
GSE: grape seed extract
GSH: glutathione
HDF: high-fat diet
p-HPEA-EDA: Decarboxymethyl ligstroside aglycone
HPLC-DAD-MS: high-performance liquid chromatography–diode array detector–mass spectrometry
HPLC-DAD-ESI/MS: high-performance liquid chromatography–diode array detector–electrospray ionization/mass spectrometry
HT: hydroxytyrosol
LC: liquid chromatography
MCP: minor polar compounds
MMP-9: matrix metalloproteinase-9
MMP: matrix metalloproteinase
MO: myeloperoxidase
NF-kβ: nuclear factor kβ
OLC: oleocanthal
OLE: oleuropein
OLEA: oleacein
OLEO: olive leaf extract enriched in OLE
ROS: reactive oxygen species
TF: tissue factor
tHT: triacetylated hydroxytyrosol.
